# Enhanced Osteoconductivity of Zirconia Implants with One-Step Femtosecond Laser Treatment Through Morphological and Chemical Modifications

**DOI:** 10.3390/jfb16040142

**Published:** 2025-04-15

**Authors:** Yuqi Li, Yanzhe Fu, Nan Li, Guanqi Liu, Jiebo Li, Jiao Wen, Jianmin Han

**Affiliations:** 1 Department of Dental Materials, Peking University School and Hospital of Stomatology, Beijing 100081, China; 2 Tianjin Key Laboratory of Oral Soft and Hard Tissues Restoration and Regeneration, School and Hospital of Stomatology, Tianjin Medical University, Tianjin 300070, China; 3 Institute of Medical Photonics, Beijing Advanced Innovation Center for Biomedical Engineering, School of Biological Science and Medical Engineering, Beihang University, Beijing 100191, China

**Keywords:** dental ceramic, hydroxylation, “one-step” functional ablation, osteogenesis, surface modification

## Abstract

Improving surface bioactivity is crucial to acquiring zirconia implants with ideal osteoconductivity. In this work, we enhanced the surface properties of zirconia implants, specifically roughness, hydrophilicity, and osteoconductivity, using a “one-step” femtosecond laser (FSL) treatment in air, deionized water, and sodium hydroxide solution. Zirconia specimens were treated in these media, and their surface morphology, chemical composition, and osteoconductivity were evaluated through various assays. The results showed that FSL treatment successfully created micro/nanoporous structures and increased roughness across all specimens. The liquid media treatment facilitated the grafting of hydroxyl (-OH) groups, significantly improving hydrophilicity. The L-NaOH group exhibited a higher hydroxyl content (28%) compared to the L-Air group (10%), reducing the contact angle significantly. Enhanced osteoblast differentiation and mineralization, along with improved gene expression, were observed in the L-Water and L-NaOH groups. In conclusion, the one-step FSL treatment developed a dual-function bioactive zirconia surface, offering an effective method for the biomedical functionalization of zirconia implants.

## 1. Introduction

Ceramics and their composites with high chemical stability and exceptional corrosion resistance have attracted considerable research and development efforts in biomedical engineering [1,2,3]. Specifically, 3 mol% yttrium oxide-stabilised zirconia (3Y-TZP) exhibits favourable mechanical properties, biocompatibility, and aesthetics, making the material highly attractive for implant fabrication, especially for dental applications [4,5,6,7,8]. Furthermore, compared with classical titanium (Ti)-based dental implants, zirconia implants demonstrated lower plaque and bleeding scores [9,10]. However, enhancing the bioactivity and increasing the osteointegration rate and degree of zirconia implants remains a major challenge [11,12].

Generally, the bioactivity of the implants is affected by their surface micromorphology and chemical composition [13,14,15]. Classical methods, including sandblasting, acid etching, and organic or inorganic coatings, have been widely utilised for zirconia im-plants to improve bioactivity by forming a porous structure, increasing the surface roughness, or modifying the surface composition. Nevertheless, sandblasting may introduce cracks and phase transitions that degrade mechanical properties. Acid etching is challenging for zirconia materials, and achieving a micro-/nanoporous surface similar to that of Ti implants is difficult. Coatings induce delamination during implantation and degradation affects the long-term stability of the implant, presenting challenges for clinical application. In addition, methods such as ion implantation, surface chemical grafting, and ultraviolet (UV) irradiation have been widely explored. Guo et al. modified the surface of zirconia using carbon and nitrogen plasma implantation, which promoted the biological activity of bone marrow mesenchymal stem cells (BMSCs) by introducing desirable nitrogen functional groups [16]. Hong et al. grafted mercapto- and amino-terminal silanes onto Y-TZP surfaces, which promoted the osteogenic differentiation and mineralization of mouse pre-osteoblast cells [17]. Han et al. demonstrated that UV irradiation was effective in the photo functionalisation of zirconia, which improved osteoblastic function [18]. Nevertheless, the processing of ion implantation and surface chemical grafting is complex, and increasing the roughness is difficult, while UV irradiation results in a yellow colour and damages the aesthetics of zirconia implants [19]. Compared with these techniques, laser ablation, which possesses remarkable flexibility and excellent controllability and has been used on various materials, offers precise and diverse surface treatments. Carvalho et al. constructed grid-like geometries on zirconia materials using a femtosecond laser (FSL), which improved metabolic activity and biological response [20]. However, these technologies focus only on the surface morphology, and a comprehensive surface treatment approach is required.

Therefore, dual-function bioactive surfaces are typically prepared using a step-by-step procedure, such as the hybrid method that combines FSL micro structuring with chemical treatments. This approach has been demonstrated to achieve dual surface functions and further enhance biological performance. Huang et al. generated micro/nanostructures on a titanium dioxide surface using FSL, followed by immersion in a sodium hydroxide (NaOH) or hydrogen peroxide or hydrothermal treatment to obtain a super hydrophilic surface [21]. Remarkably, the simultaneous alteration of surface micromorphology and chemical composition by laser treatment has garnered significant attention. Mazur et al. demonstrated that the ambient medium significantly influences the quality of laser treatments [22]. In the cases of laser ablation in liquid, various chemical reactions may occur between the ablated specimens and ambient liquid molecules [23]. For instance, a hierarchical micropattern covered with calcium phosphate was constructed when a Ti implant was placed in a hydroxyapatite suspension medium and irradiated with FSL, which expedited early osseointegration [24]. Thus, as a promising “one-step” surface treatment approach, the technique combines morphological change and designable function using laser ablation in certain media. However, no such studies have been conducted on zirconia surfaces.

In this study, we first employed a one-step functional ablation method in which 3Y-TZP specimens were ablated using FSL in various media (air, deionized [DI] water, NaOH solution). The surface morphology, roughness, and chemical composition of the zirconia specimens after FSL treatment were evaluated, and the impact of the ablation process on the surface wettability was analysed. Additionally, the biocompatibility and osteogenic potential of the functionally ablated zirconia were evaluated by analysing cell adhesion, proliferation, and osteogenesis in vitro. This study focused on the application of the one-step functional ablation method to zirconia, intending to improve its biological activity and provide guidelines for the surface modification of ceramic implants. By accelerating osseointegration, this approach improves early implant stability. Moreover, the sustained bioactive properties would contribute to long-term implant success. The synergistic effects of these advantages not only extend implant longevity, but also shorten patient recovery time and enhance overall clinical efficacy.

## 2. Materials and Methods

### 2.1. Preparation of Zirconia Specimens

The ZrO_2_ powder (ZrO_2_ + HfO_2_ 94.35 ± 0.30 wt.%; Y_2_O_3_ 5.65 ± 0.30 wt.%; Al_2_O_3_ 0.25 ± 0.15 wt.%; impurities SiO_2_ + Fe_2_O_3_ + Na_2_O + CaO + TiO_2_ ≤ 0.1 wt.%, HSY-3FSD-J) for 3Y-TZP specimens manufacture was provided by DAIICHI KIGENSO KAGAKU KOGYO CO, Ltd., Osaka, Japan. The disc specimens (Φ14.5 × 1.2 mm) was prepared by dry pressing at 167 MPa for 5 min. The green body was sintered at 1450 °C for 2 h with a heating rate of 30 °C/min. Then, all sintered specimens were soaked in acetone, anhydrous alcohol, and DI water sequentially and cleaned in an ultrasonic cleaner for 20 min. Ethics approval was not required for this in vitro study.

### 2.2. Femtosecond Laser Treatment

**Figure 1:**
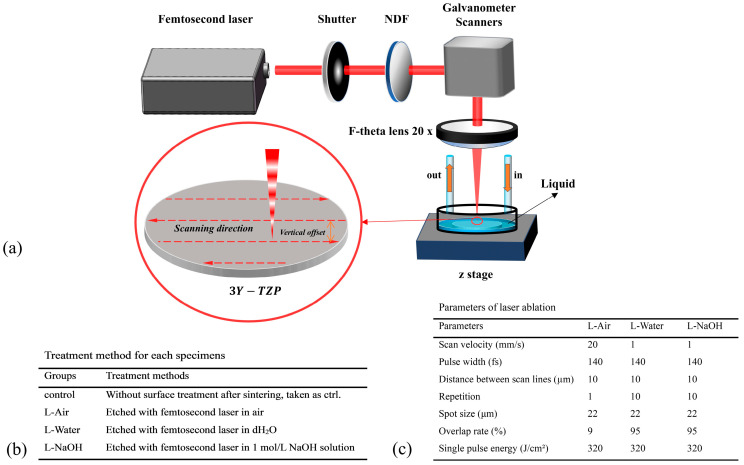
(**a**) Experimental setup for the laser ablation of 3Y-TZP in DI water or NaOH solution; (**b**) treatment method for each specimens; (**c**) laser ablation parameters.

As illustrated in the schematic of the experimental setup in Figure 1a, an ultrafast Ti: Sapphire amplifier (Phidia C, UpPek Solutions, Bohemia, NY, USA) was employed for ablation. The laser was operated in the direct writing mode with a centre wavelength of 790 nm, pulse width of 120 fs, repetition rate of 1 kHz, and laser power of 1 W. The laser was focused on the sample surface through an f-theta lens (20×) and scanning was facilitated using a two-axis galvanometer scanner. The 3Y-TZP specimens were divided into four groups, and the details of the treatment methods are demonstrated in Figure 1b. When laser ablation was performed in DI water or a 1 mol/L NaOH solution, the liquid surface was maintained 1 mm above the 3Y-TZP specimen surface. Droplet-based microfluidics were utilised to maintain the liquid level stability, and the flowing DI water or NaOH solution could simultaneously remove the ablation debris.

### 2.3. Surface Morphology Characterisation

The surface micro-morphology was observed by a scanning electron microscope (SEM, S4800, Hitachi, Japan) with an energy of 15 kV in secondary electron mode. The surface roughness was evaluated in terms of Sa (Arithmetic Mean Height) and Sq (Root Mean Square Height) by a white light interferometer (WLI, ATOMETRICS, Shenzhen, China) under 10× magnification glass. The phase of the 3Y-TZP specimens were characterised on an X-ray diffractometer (Bruker AXS, D8 Advance, Karlsruhe, Germany) using Cu-Kα radiation (1.54 Å) with 2θ ranging from 25° to 35° at a rate of 2°/min with a scanning step of 0.02°.

### 2.4. Surface Chemical Composition and Wettability Characterisation

The surface chemical components were determined by X-ray photoelectron spectroscopy (XPS, Thermo Kalpha, Waltham, MA, USA) using Al-Kα radiation (1486.6 eV) as the source. The binding energies were calibrated using the C1s at 284.8 eV. Three samples were analysed for each group.

The wettability was assessed using a contact angle (CA) meter (DSA100E, KRUSS, Hamburg, Germany), with the CA calculated from the measurement of 2 μL DI water on the surface of the 3Y-TZP specimens. Five samples were analysed in each group, two regions were selected for measurement on each specimen.

### 2.5. Biocompatibility Assessment In Vitro

#### 2.5.1. Cell Culture

Human BMSCs (hBMSCs: ATCC, Manassas, VA, USA) were cultured in alpha-modified minimum essential medium (α-MEM, Gibco, Carlsbad, CA, USA) at 5% CO_2_ and 100% humidity. For cell proliferation, the proliferation medium contained 10% foetal bovine serum (FBS, Gibco, Sydney, Australia) and 1% penicillin/streptomycin (Gibco). For osteogenic induction, the osteogenic medium contained 10% FBS, 1% antibiotics, 10 nM dexamethasone, 200 μM ascorbic acid, and 10 mM β-glycerophosphate. The fourth- to sixth-generation cells were seeded on the 3Y-TZP specimens, which were placed in 24-well plates at a density of 3 × 10^4^ cells/well. All in vitro studies included three specimens, with each specimen having three replicate measurements.

#### 2.5.2. Cell Morphology and Adhesion

For the cell morphology assay, hBMSCs were incubated on the surface of the 3Y-TZP specimens for 24 h and then rinsed with phosphate-buffered saline (PBS) three times. Cells were fixed with 2.5% glutaraldehyde (*v*/*v*) for 30 min and dehydrated in 50%, 75%, 90%, and 99% ethanol for 15 min. The samples with fixed cells were then coated with a thin layer of gold for SEM observation.

The hBMSCs were cultured on the 3Y-TZP specimens a certain number of times and rinsed thrice with PBS. Cells were fixed with 4% paraformaldehyde for 30 min at room temperature and permeabilised with 0.1% Triton X-100 in phosphate buffer for 5 min. After rinsing with PBS, the cells were stained with 5 μg/mL rhodamine phalloidin (Solarbio Science & Technology Co., Ltd., Beijing, China) for 40 min and 4′,6-diamidino-2-phenylindole (DAPI) (Sigma, St. Louis, MO, USA) for 5 min, and then observed using laser scanning confocal microscopy (TCS-SP8, Leica, Vizsla, Germany).

#### 2.5.3. Cell Proliferation

For the cell proliferation, cell counting kit-8 (CCK8; Dojindo Laboratories, Kumamoto, Japan) was employed to determine the number of cells attached to the 3Y-TZP specimens after incubation for 1, 3, and 5 d (named d1, d3, and d5, respectively). The medium was refreshed with the counting reagent, and the cells were incubated for 2 h at 37 °C. A microplate reader (SoftMax Pro, Molecular Devices, Jose, CA, USA) was utilised to measure spectrophotometric absorbance at a wavelength of 450 nm. Each group was tested in triplicate.

### 2.6. In Vitro Osteogenic Potential Assessment

Alkaline phosphatase (ALP) activity was determined to estimate early osteogenic differentiation. After 7 d of incubation, the hBMSCs were fixed with 99% ice-cold ethanol for 30 min, and a BCIP/NBT ALP colour development kit (Beyotime Biotechnology, Shanghai, China) was used for staining for 30 min, following the manufacturer’s instructions.

Alizarin red S (ARS) staining was used to estimate the formation of mineralised nodules. After 21 d of incubation, the hBMSCs were fixed in 4% paraformaldehyde for 30 min at room temperature and then treated with ARS solution (2%, pH 4.2, Sigma-Aldrich, Poole, UK) for 20 min.

Total cellular RNA was extracted from hBMSCs cultured in osteogenic medium for 7 and 14 d using a Cell Total RNA Isolation Kit (Foregene, Chengdu, China). Complementary deoxyribonucleic acid was synthesised using the Prime Script RT Master Mix (Takara Biotechnology, Shiga, Japan). Reverse transcription-quantitative polymerase chain reaction (PCR) was performed using the SYBR Green Master Mix (Roche Applied Science, Mannheim, Germany) on an ABI Prism 7500 Real-Time PCR System. Gene expression was normalised to that of the reference gene, glyceraldehyde-3-phosphate dehydrogenase (GAPDH). Primer sequences for human GAPDH, ALP, osteocalcin (OCN), osterix (OSX), and runt-related transcription factor 2 (Runx2) are listed in Table 1.

Data are expressed as mean ± standard deviation (SD). Normality was assessed prior to statistical analysis. Statistical analyses were conducted using GraphPad Prism 9.5.0 software (GraphPad Software, San Diego, CA, USA), and intergroup comparisons were performed using the independent t-test. A *p*-value < 0.05 was considered statistically significant.

## 3. Results

### 3.1. Surface Morphology of the 3Y-TZP Specimens

**Figure 2:**
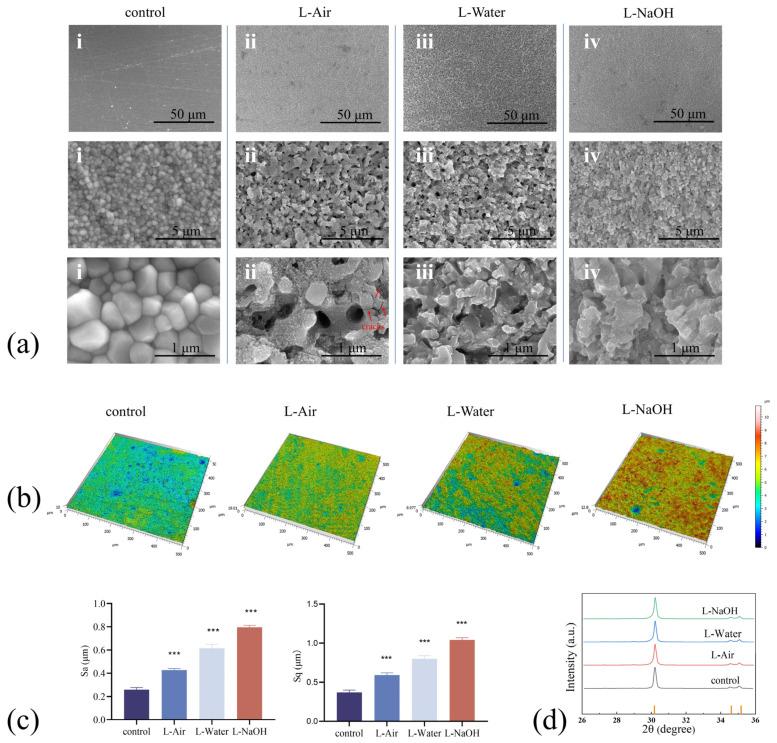
SEM images of surface morphology for (**a**) (i) control specimen, (ii) L-Air, (iii) L-Water, and (iv) L-NaOH; (**b**) 3D surface morphology images of the 3Y-TZP specimens; (**c**) roughness of the 3Y-TZP specimens; (**d**) XRD patterns of the 3Y-TZP specimens. Data are means ± SD; *** *p* < 0.001 compared to the control group.

The results of the surface morphology analysis with various FSL treatments are demonstrated in Figure 2a. The surface structure of the control specimen without laser treatment was extremely smooth, with a relative density of 99.6% (Figure 2a(i)). Conversely, the ablated specimen exhibited an altered surface morphology and the original grain structure completely collapsed. A concave–convex structure with pores between 100 and 500 nm was created after ablation in air. Microcracks appeared near the pores, and nanoparticles were stacked on the surface (displayed in Figure 2a(ii)). No cracks or stacked nanoparticles were observed in the L-Water specimen (illustrated in Figure 2a(iii)). As for the L-NaOH specimen, the smooth surface was transformed into a structure of sub-micro protrusions using the laser treatment (displayed in Figure 2a(iv)) [25,26].

Three-dimensional morphology images of the specimens before and after laser ablation are presented in Figure 2b, along with the roughness results (Sa and Sq) illustrated in Figure 2c. The control specimen was smooth and had the lowest roughness of 0.26 μm (Sa) and 0.37 μm (Sq). The roughness of the specimen was increased following laser treatment, with the L-NaOH specimen exhibiting the coarsest surface measuring 0.80 μm (Sa) and 1.04 μm (Sq). We noted that the L-Air specimen had less roughness than that observed with the liquid specimen, which could be attributed to the scanning repetition and total energy differences. XRD patterns displayed in Figure 2d demonstrate that the as-sintered 3Y-TZP specimen was composed of the t-phase. Considering the short action period and minimal thermal effect of FSL, laser treatment does not contribute to phase transformation [20,27].

### 3.2. Surface Chemical Composition and Wettability Modified by Laser Ablation

**Figure 3:**
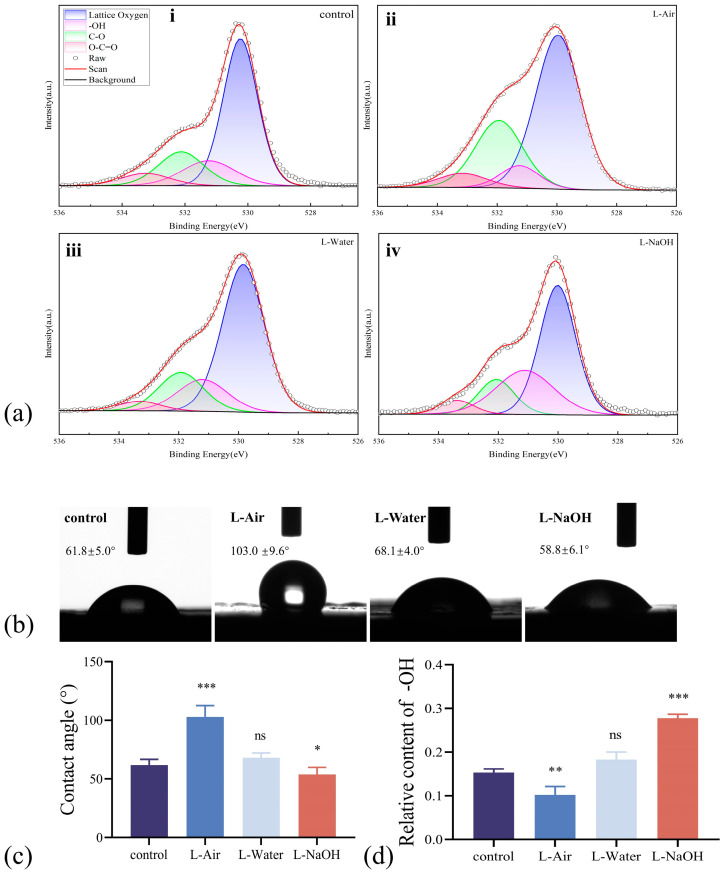
(**a**) High-resolution XPS spectra of O1s and their deconvolution; (**b**) the image of water contact; (**c**) the relative content of the -OH on 3Y-TZP surfaces; (**d**) water contact angle. Data are means ± SD; * *p* < 0.05, ** *p* < 0.01, *** *p* < 0.001, ns indicate no significance compared to the control group.

As stated previously, surface structures and chemical compositions manage wettability. To further illuminate the effects of laser ablation and the treatment medium, the surface chemistry was analysed. The O 1s peaks of the 3Y-TZP specimens XPS spectra, which were deconvoluted into four peaks, are displayed in Figure 3a. The peak near 530 eV was attributed to lattice oxygen, the peak near 531.2 eV to hydroxyl (-OH) groups, the peak near 532 eV to C-O bonds, and the peak near 533 eV to O = C-O groups, respectively [28,29]. The OH content of the control specimen consisted of 15% -OH groups. The intensity of the peak representing -OH after laser ablation in the air group decreased to 10% (demonstrated in Figure 3a(ii). After treatment in liquids, the intensity of -OH groups increased, with the percentage of -OH reaching 18% in L-Water and 28% in L-NaOH (illustrated in Figure 3a(iii,iv).

The wettability of the water drop on the 3Y-TZP specimen was examined, and images of the CA are displayed in Figure 3b. The control specimen exhibited hydrophilicity and a CA of approximately 61.8°. Nevertheless, the L-Air specimen with the lowest -OH content exhibited a CA of 103.0°, indicating a hydrophobic surface. In contrast, the L-Water and L-NaOH specimens exhibited hydrophilic properties, with CA values of 68.1° and 58.8°, respectively. For the specimens with rough surfaces, air was trapped in the grooves. Droplet diffusion and spread are constrained when the diameter is large or comparable to the size of the grooves [30]. After laser ablation, micro/nanostructures of concave–convex pores or protrusions formed on the zirconia specimen, affecting the wettability and decreasing the CA of the L-Air specimen. For the L-water and L-NaOH specimens, the wettability may have worsened due to the morphology change in the surface. A reduced CA was obtained when the wettability was improved by the terminal group -OH [31]. Thus, we speculate that the grafted -OH terminal group counterbalances the negative impact of the microstructure sharply.

### 3.3. Biocompatibility Assessment In Vitro

**Figure 4:**
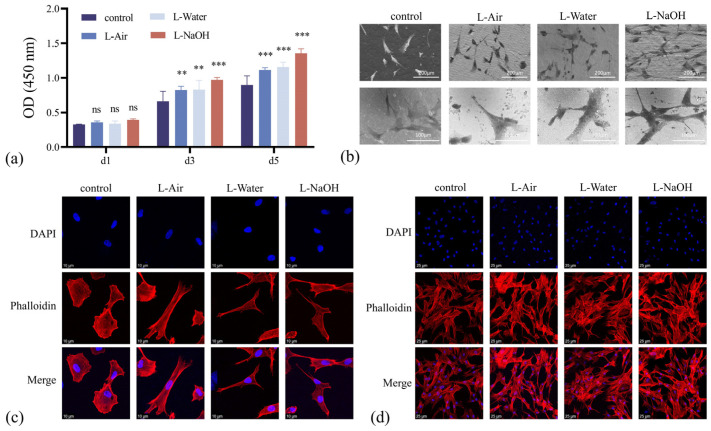
(**a**) CCK8 assay of adhering hBMSCs on four different surfaces after 1, 3, and 5 d of culture; (**b**) SEM observation of hBMSC morphology after 24 h of culture at 200× and 400× magnification; (**c**) LSCM micrographs of cellular morphology on four groups specimens after 4 h of inoculation; (**d**) LSCM micrographs of cellular morphology on four groups specimens after 24 h of inoculation. Data are means ± SD; ** *p* < 0.01, *** *p* < 0.001, ns indicate no significance compared to the control group.

Cell proliferation capacity was quantitatively determined using the CCK8 assay, and the optical density (OD) values of all specimens continued to increase over the culture period (Figure 4a). The OD values for each specimen were similar, indicating a comparable number of cells were inoculated on 1 d. The specimens that received laser treatment had greater OD values after 3 and 5 d, revealing that a significant number of hBMSCs were attached to the surface. Compared with the structure in the control specimen, the porous structure in the L-Air specimens expanded the surface area and was accessible for cell attachment. The cell proliferation capacity was further improved for the specimens ablated in a liquid with a porous structure and grafted -OH. After 5 d, the L-NaOH specimen presented an exceptionally high OD value.

The morphologies of hBMSCs cultured on different specimens for 24 h are presented in Figure 4b. Cells in the control specimen exhibited a polygonal shape with unclear pseudopod extension. Although the cells on the laser-treated specimens also demonstrated a polygonal shape, pseudopod expansion was considerably noticeable. Immunofluorescence staining images reflected the cell adhesion of hBMSCs cultured on different specimens for 4 and 24 h (Figure 4c,d). Nuclei were stained with DAPI (blue), and the actin cytoskeleton with FITC (phalloidin, red). Additionally, Figure 4c displays that after inoculation for 4 h, the cells on the control specimen surface were flattened. In comparison, the 3Y-TZP specimen treated with the laser exhibited distinct cells with a stretched actin cytoskeleton and slender pseudopods. After 24 h, the cells in Figure 4d revealed a dense actin cytoskeletal network. In addition, the proliferating hBMSCs completely covered the surfaces of the laser-treated specimens, with the cells in direct contact with each other. Hence, the micro/nanostructure generated by laser ablation improved the biocompatibility of the 3Y-TZP specimen, and the grafted OH further enhanced the rate of cell proliferation. The highest cell viability was observed for the L-NaOH specimen, suggesting that the hydroxylation-treated 3Y-TZP specimen was advantageous for cell proliferation.

### 3.4. In Vitro Osteogenic Potential Assessment

**Figure 5:**
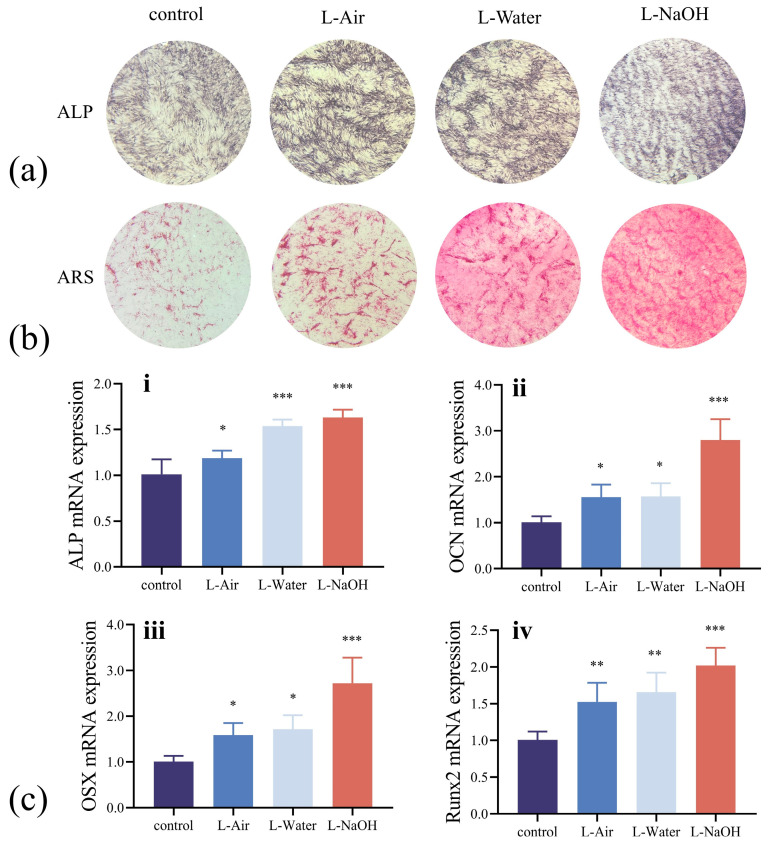
(**a**) ALP staining of hBMSCs cultured after 7 d of osteogenic induction; (**b**) ARS and mineralization assay of hBMSCs cultured after 21 d of osteogenic induction; (**c**) osteogenic gene expression of hBMSCs after 14 d of osteogenic induction. Data are means ± SD; * *p* < 0.05, ** *p* < 0.01, *** *p* < 0.001 compared to the control group.

We also examined the in vitro osteogenic potential of the four groups of the 3Y-TZP specimens. ALP was used to analyse early osteogenic differentiation, with Figure 5a displaying the stained specimens. The laser-ablated specimens demonstrated significant ALP staining after 7 d. In particular, the L-NaOH specimen stained dark blue and exhibited the highest osteogenic induction activity. Extracellular matrix mineralisation was reflected by ARS staining, which was used to examine the later stages of osteogenic differentiation. Numerous calcium deposits, in the form of nodules, were observed on the surfaces of the laser-treated specimens, as demonstrated in Figure 5b. The L-NaOH specimen also exhibited the most intense staining and osteogenic potential, which was consistent with the ALP results. Osteogenic potential has been explored at the molecular level by testing the messenger RNA expression of several genes related to osteogenesis. The expression levels of ALP, OCN, OSX, and Runx2 genes are represented in Figure 5c. OCN is a major non-collagenous protein of the extracellular matrix synthesised by mature osteoblasts, OSX is a zinc finger-containing transcription factor essential for the maturation of osteoblasts, and Runx2 is an osteoblast-specific transcription factor required for the induction of osteoblast differentiation that regulates the expression of other osteogenesis-related genes, such as ALP, OCN, and OSX [32,33,34,35]. Compared to the untreated 3Y-TZP specimen, the L-Air specimen with a porous structure exhibited a higher level of cell proliferation. Furthermore, the specimen ablated in NaOH solution (presented in Figure 5c(ii,iii) exhibited a significantly high expression of OSX and OCN, indicating accelerated osteoblast differentiation due to the grafted -OH.

## 4. Discussion

The osteoconductivity of the dental implants depends on their surface characteristics. In particular, the composition, roughness, porous structure, and hydrophilicity of the implant surface govern cell adhesion, proliferation, and differentiation [36,37]. The surface micro/nanostructure of 3Y-TZP dental implants may promote cell adhesion and achieve mechanical interlocking [38]. Appropriate physical and chemical surface properties of zirconia implants can enhance their biological activity.

In the present study, the surface morphology of zirconia was modified by laser ablation, and a microstructure consisting of concave–convex pores or protrusions was formed. The treatment medium and incremental scan repetition increased the roughness of the 3Y-TZP. Additionally, the coarsest structure was generated on the L-NaOH specimen. Notably, the roughness of the L-NaOH specimen was nearly at the ideal level (Sa of ~1 μm), which was considered optimal for promoting bone in-growth [8,39]. Additionally, the L-NaOH specimen protrusions were considerably uniform and free of fractures, which prevented stress concentration and decreased the possibility of degradation of the mechanical properties [40,41].

**Figure 6:**
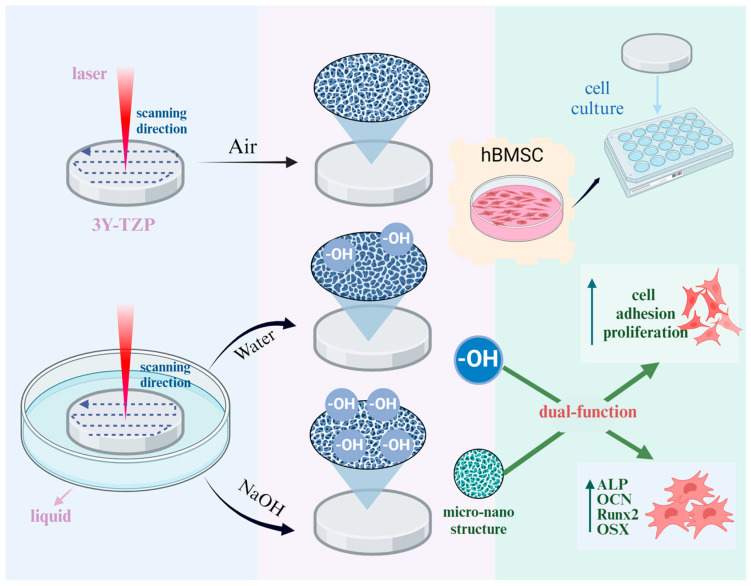
Schematic illustration depicts the dual functionality of laser-ablated 3Y-TZP in various media. The grafting of -OH groups onto the surface micro-morphology enhances the proliferation, adhesion, and osteogenic differentiation of hBMSCs while also increasing the expression of relevant genes.

Typically, wettability is governed by surface hydrophilic groups such as -OH. The CA test of the L-Air specimens demonstrated that the wetting mode transformed from a Wenzel to a Cassie-dominated state due to the micro/nanostructure [28], and the wettability decreased as the air trapped in the microstructures prevented the spread of the water droplets. The wetting mode transformation may have occurred in the L-Water and L-NaOH specimens whose surfaces were modified by FSL ablation. In fact, abundant -OH was grafted onto the zirconia surface after ablation in a solution of NaOH or DI water (Figure 6), which weakened the impact of roughness. The CA values of the L-water and L-NaOH specimens were significantly lower than those of the control and L-Air specimens [42,43]. The impact of -OH was further confirmed using the L-NaOH specimen with the highest -OH content and minimal CA value. We observe that zirconia specimens with comparable wettability and roughness may exhibit comparable biological behaviour with L-NaOH, which is challenging to achieve by direct laser treatment in air. Further research is anticipated to yield more techniques for the dual-functional surface treatment.

According to an in vitro test using hBMSCs, the 3Y-TZP specimen, which had a smooth surface, exhibited a certain level of bioactivity in which the cultured cells could proliferate and undergo osteogenic differentiation. The L-Air specimen with a rough morphology had elevated surface energy and provided greater adhesion sites, which enhanced the initial protein adsorption and facilitated cell interactions [44]. Furthermore, hydrophilic groups such as -OH, –COOH, and -CONH_2_ can alter wettability and enhance protein adsorption [45]. The OH groups in the L-Water and L-NaOH specimens demonstrated promising osteoblast differentiation and mineralisation behaviours in the hBMSCs. Gene expression was highest in the L-NaOH specimen, which had the highest -OH and protrusion structures.

Hence, simultaneous functionalisation of morphological alteration and terminal group modification was achieved via a one-step FSL treatment supported by a suitable medium. However, its application is still limited by processing efficiency and the need for complex control programs for profiled implant surfaces. To further enhance this approach, future research should focus on adapting the one-step method to economical nanosecond pulse lasers, optimising treatment parameters, and evaluating the in vivo performance of laser-treated zirconia. Additionally, adjusting the type and concentration of the solution and grafting more terminal groups to the -OH sites could further refine the functionalization process, expanding its potential for clinical applications.

## 5. Conclusions

Taken together, we established a micro/nanoporous and hydrophilic surface for zirconia implants using “one-step” laser ablation in a liquid solution. In particular, the specimen that emerged in the NaOH solution (L-NaOH) exhibited uniform microprotrusions and was grafted with abundant hydrophilic terminal group -OH. The adhesion and proliferation of hBMSCs were enhanced by the rough surface created by laser ablation. Moreover, OH facilitated protein adsorption and surface wettability, which improved osteogenesis and enhanced gene expression. Furthermore, the -OH effect increased with increasing concentration, and the L-NaOH sample exhibited the highest level of bioactivity. This study provides an effective and reliable strategy for improving the bioactivity of zirconia implants.

## Figures and Tables

**Table 1:** Primer sequences for RT-PCR.

Target Gene	Forward Primer (5′-3′)	Reverse Primer (5′-3′)
GAPDH	GGAAGCTTGTCATCAATGGAAATC	TGATGACCCTTTTGGCTCCC
ALP	CTCCTCGGAAGACACTCTGACC	CTGCGCCTGGTAGTTGTTGTG
OCN	CCTCACACTCCTCGCCCTATT	CCGATGTGGTCAGCCAACTC
OSX	TTTACCCGAAGCGACCACC	GAGTGATTGGCAAGCAGTGGTC
Runx2	CTACTATGGCACTTCGTCAGGAT	ATCAGCGTCAACACCATCATT

## Data Availability

The original contributions presented in this study are included in the article. Further inquiries can be directed to the corresponding authors.

## Abbreviations

The following abbreviations are used in this manuscript:

ALP	Alkaline phosphatase
ARS	Alizarin red S
DI	Deionized
FSL	Femtosecond lase
hBMSCs	Human bone marrow mesenchymal stem cells
NaOH	Sodium hydroxide
hBMSCs	Human bone marrow mesenchymal stem cells
OCN	Osteocalcin
OD	Optical density
OSX	Osterix
PBS	Phosphate-buffered saline
Runx2	Runt-related transcription factor 2
UV	Ultraviolet
3Y-TZP	3 mol% yttrium oxide-stabilised zirconia
